# Malaria treatment-seeking behaviour and related factors of Wa ethnic minority in Myanmar: a cross-sectional study

**DOI:** 10.1186/1475-2875-11-417

**Published:** 2012-12-14

**Authors:** Jian-Wei Xu, Qi-Zhang Xu, Hui Liu, Yi-Rou Zeng

**Affiliations:** 1 Yunan Institute of Parasitic Diseases, Puer, 665000, China; 2 People’s Hospital of Taikang County, Henan Province, China; 3 Mengmao County Hospital, Shan Special Region II, Myanmar

**Keywords:** Malaria, Treatment-seeking behaviour, Wa ethnic, Household survey

## Abstract

**Background:**

In Southeast Asia, data on malaria treatment-seeking behaviours and related affecting factors are rare. The population of the Wa ethnic in Myanmar has difficulty in accessing formal health care. To understand malaria treatment-seeking behaviour and household-affecting factors of the Wa people, a cross-sectional study carried out in Shan Special Region II, Myanmar.

**Methods:**

The two methods, questionnaire-based household surveys to household heads and in-depth interviews to key informants, were carried out independently. The proportion of treatment-seeking patterns was calculated. Logistic regression was used to determine affecting factors of treatment-seeking. Qualitative data were analysed by using Text Analysis Markup System.

**Results:**

Overall, 87.5% of the febrile population sought treatment, but only 32.0% did so within 24 hours. The proportion accessing the retail sector (79.6%) was statistically significant higher (P<0.0001) than accessing the public sector (10.6%). Multivariable logistic regression analysis identified family income, distances from a health facility, family decision and patient characteristics being independently associated with delayed malaria treatment.

**Conclusion:**

Malaria treatment-seeking behaviour is not appropriate, and affecting factors include health service systems, social and cultural factors in Wa State of Myanmar.

## Background

There were an estimated 216 million episodes of malaria and 655,000 malaria deaths worldwide in 2010 [1]. Malaria is a major cause of poverty and slows economic growth by up to 1·3% per year in endemic countries [2]. Early diagnosis and effective treatment of all malaria cases is an essential component to reduce the burden of malaria. This requires appropriate infrastructure and resource, and also active engagement and participation of communities [3]. Data on malaria treatment-seeking behaviours and household-affecting factors are rare in Southeast Asia [4,5]. The Wa ethnic minority lives across the China-Myanmar border and its total population is around 1.2 million (740,000 in Myanmar side, and 460,000 in China) [6]. Malaria control among the population of ethnic minorities is being challenged by treatment-seeking behaviours and accessibility to health service [6]. In Myanmar, Wa people mainly live in Shan Special Region II (locally called Wa State or UWSA territory). Malaria is one of the major public health problems among the population. An active detection found 60% (270/453) of parasite rates among febrile patients and of them 90.74% (245/270) was *Plasmodium falciparum*[7]. In Wa State, most residents have difficulty in accessing formal health care. Data and information concerning malaria treatment for them are insufficient. This investigation could assist in understanding treatment-seeking behaviour and related household factors.

## Methods

### Study area and period

**Figure 1:**
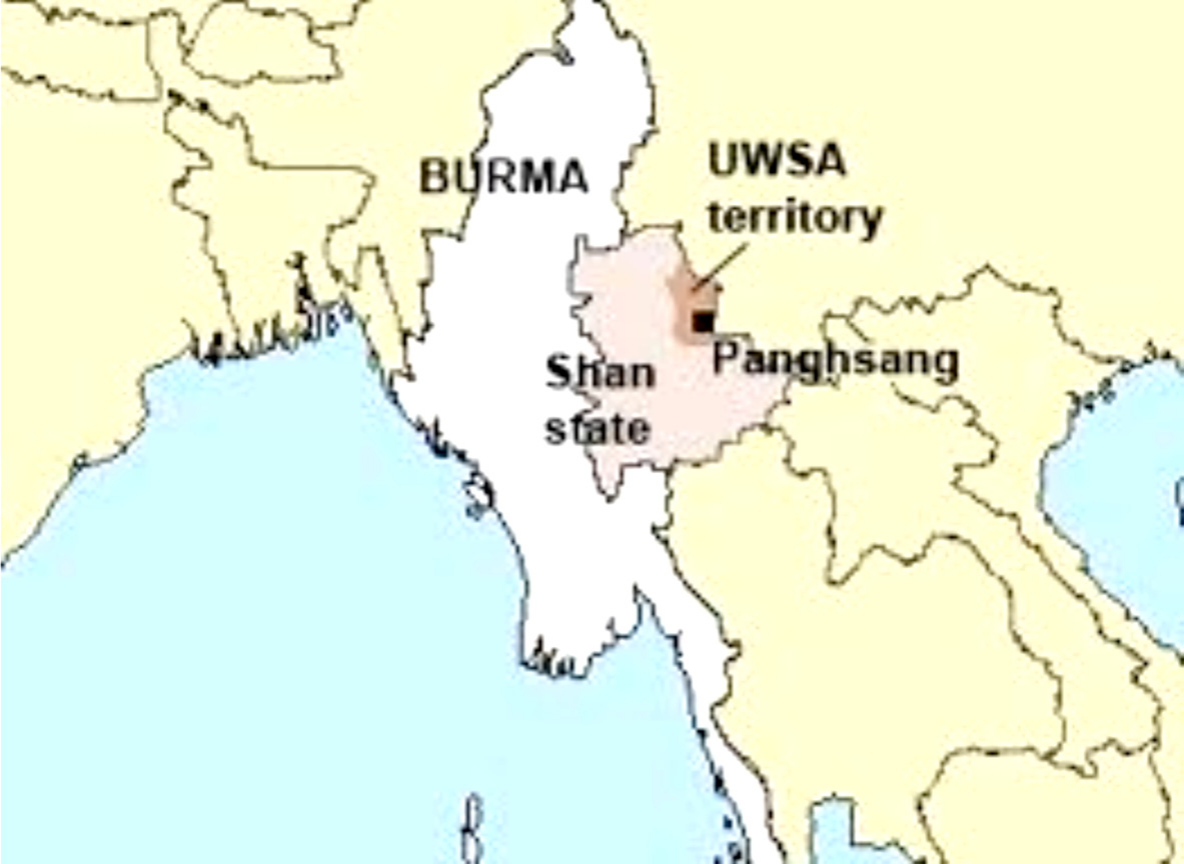
The study site, Shan Special Region II (UWSA territory), Myanmar (Burma).

A cross-sectional study was conducted between 1 October and 31 December 2009 in Gelongba and Mandong districts, Mengmao County, Wa State (Figure 1). The two districts in Salween River Valley were purposely selected based on their malaria endemicity and prevalence. This area experiences year-round malaria transmission with a peak during the rainy season from September to November. Health care is provided by a community health centre, two NGO health posts by Aide Medical International (AMI) and two private clinics. Drugs are provided by two drug shops and market stalls. All these facilities of health care and pharmacy are located the two main villages: Gelongba and Mandong.

### Household survey design and implementation

The study population included people who had signs/symptoms of malaria in the previous two weeks. The investigation was carried out by household survey (HHS) as a quantitative method and semi-structured in-depth interviews (SDI) to key informants as the qualitative [8,9].

The data collection tools (questionnaires and interview guidelines) were developed in Chinese because Wa language is only a speaking language. One of researchers who can understand both Wa and Chinese Language conducted the interviews in Wa language and then filled out the questionnaires in Chinese. The sampling frame for the survey covered all 64 villages of the districts of Gelongba and Mandong, which have an approximate total population of 18,940. Households were the units of sampling. A sample size of 350 households was required by using desired 5% of precision, estimated 35% of febrile patients in the previous two weeks who sought treatment within 24 hours and 95% of confidence limits. A household was defined as all those eating from the same cooking pot. Sampling of households was restricted to those with a fever patient in the previous two weeks.

The survey started from Gelongba, one of two main villages. The first household was randomly selected. The researchers visited the subjects village by village and house by house. A household with a fever patient in the previous two weeks was selected for the survey, until the desired sample size was reached. The household head was selected on behalf of the entire household to answer the questionnaires. The questionnaire included structured questions on respondent and household characteristics, details of the treatment-seeking behaviours and malaria-related knowledge [10,11]. In order to reduce recall bias, households without fever in the last two weeks were just interviewed for socio-demographic characteristics but for treatment-seeking and other related factors. Details of the youngest one were collected if there was more than one person who had had fever in a household in last two weeks.

To collect information in detail for exploring malaria treatment-seeking, the SDIs were conducted in the same villages sampled for HHS. According to the recommendation of household heads, 23 village heads and 13 village health workers were selected as key informants for interview. The issues discussed with key informants were local health service, people’s perception of malaria, treatment-seeking behaviours and related socioeconomic status [12].

All health facilities were visited and their staffs were interviewed on health service in the two districts. An outlet survey of anti-malarial drugs was also conducted and the survey included interviewing medical sellers on their knowledge of drugs.

### Data analysis

Both quantitative and qualitative data analysis was carried out by two senior researchers (J-W & H L). Quantitative Data were first checked manually for completeness and then double-entered and validated in EpiData version 3.1 [13]. Epi Info 2000 was used for data processing and analysis. Bivariate analysis between dependent and independent variables was performed using binary logistic regression. To control the effect of confounding variables, multivariate logistic regressions were done. Adjusted OR and 95% CI were used to interpret the findings. Qualitative data were analysed by using Text Analysis Markup System (TAMS). The data were encoded on the basis of emerging themes and a codebook was progressively elaborated. Trends in the data were identified by producing matrices allowing for combination and comparison of information from the different key informants.

### Ethical approval

According to the Helsinki Declaration, ethical approval for the study was granted by the Ethics Committees of Yunnan Institute of Parasitic Diseases, China. The purpose of the study was explained to the local health authority and the study participants and informed consent was obtained.

## Results

### Sociodemographic characteristics of respondents

A total of 718 households were visited and a total of 3,678 people lived in the households. The mean family size was 5.8 (range 1–13) persons per household. 369 heads of households with fever patients in previous two weeks were interviewed. Sociodemographic characteristics were similar (P>0.05) between households visited and households with fever patients, except resident altitude. This might be because people living at low altitude contract malaria more readily (Table 1). The mean age of respondents was 35.6 (SD ± 13.5) years, 240 (65.0%) were male. Most households mainly live on growing dry field rice, and a few households have a member working in rubber plantation. The mean age of the 36 key informants was 32.7 (SD ± 17.5) years, and all 23 village heads were male, seven village health workers were male and five female.

**Table 1:** Socio-demographic characteristics of respondents in Gelongba and Mandong Districts, Shan Special Region II, Myanmar

	**All households visited (n=718)**	**Households with fever patients in previous 2 weeks (n=369)**	**P-value**
**Sex**			
Male	478 (66.6%)	240 (65.0%)	
Female	240 (33.4%)	129 (35.0%)	0. 6615
**Age (years)**			
≤ 30	405 (56.4%)	207 (56.1%)	
>30	313 (43.6%)	162 (43.9%	0.9739
**Educational status**			
Illiterate	701 (97.6%)	357 (96.3%)	
Read and write	17 (2.4%)	12 (3.3%)	0.5105
**Annual average income (US$)**			
≤ 100	385 (53.6%)	191 (51.8%)	0.6048
101-200	254 (35.4%)	134 (36.3%)	0.8112
>200	79 (11.0%)	40 (10.8%)	0.9831
**Family size of the household**			
≤ 5	376 (52.4%)	183 (49.6%)	
> 6	342 (47.6%)	186 (50.4%)	0.4223
**Residence**			
Altitude < 800 m	148 (20.6%)	116 (31.4%)	
Altitude ≥ 800 m	570 (79.4%)	253 (68.6%)	0.0001
**Malaria knowledge**			
Knew mosquitoes caused malaria	228 (31.8%)	114 (30.9%)	0.8256
Knew fever as symptom of malaria	508 (70.8%)	261 (70.7%)	0.9494
Did not know correct malaria knowledge	212 (29.5%)	92 (24.9%)	0.1268
**Distance from health facilities or drug shops**			
≤ 3 km	159 (22.1%)	74 (20.1%)	
> 3 km	559 (77.9%)	295 (79.9%)	0.4732
**Family decision**			
Wife or co-decision	139 (19.4%)	75 (20.3%)	0.7652
Husband	545 (75.9%)	283 (76.7%)	0.8307
No respondence	34 (4.7%)	11 (3.0%)	0.2247
**Sex of fever patients**			
Male	-	227 (61.5%)	-
Female	-	142 (38.5%)	-
**Age category of fever patients**			
≤ 15	-	41 (11.1%)	-
16 - 50	-	276 (74.8%)	-
> 50	-	52 (14.1%)	-

### Treatment-seeking behaviours

Overall 87.5% (323) of the febrile sought treatment; 32.0% (118) sought it within 24 hours, 6.5% (24) within 24–48 hours and 49.2% (181) after 48 hours. Of the 323 fever patients who sought treatment, 79.6% (257) went to the retail sector (drug peddlers, shops and market stalls); 8.4% (31) sought from village health workers (the lowest level public health facility); 2.2% (eight) went to community health centres; 7.3% (27) sought advice or treatment from other sources such as a traditional healer, a friend or relative (Table 2). The proportion accessing the retail sector was statistically significantly higher than accessing the public sector (P<0.0001). Of the 46 who stayed at home, 73.9% (34) already had drugs in the house; 67.4% (31) sought help from supernatural spirit. The proportion of fever patients who received laboratory-based diagnosis was low (20.08% for microscopy and 8.1% for RDT, respectively) (Table 2). Of 104 febrile who received laboratory-based diagnosis, 7.7% (eight) received it at the community health centre and 92.3% (96) in outreach service of NGOs. Of the 257 who sought treatment in the retail sector, 65.8% (169) received under-dosed paraquin; 13.0% (42/323) took herbs and 3.4% (11/323) took only a febrifuge, such as paracetamol.

**Table 2:** Malaria treatment-seeking behaviour of fever patients in previous two weeks in Gelongba and Mandong Districts, Shan Special Region II, Myanmar

		**Number**	**Percent (95%CI)**
**Time between onset of illness and treatment sought**		
24 hours	118	32.0 (27.2-37.0)
25-48 hours	24	6.50 (4.2-9.5)
> 48 hours	181	49.2 (43.8-54.3)
Never sought treatment	46	12.5 (9.3-16.3)
**Place where first advice and treatment sought**		
Drug peddlers	178	48.2 (43.0-53.5)
Drug shops and market stalls	79	21.4 (17.3-26.0)
Community health centre	8	2.2 (0.9-4.2)
Village health workers	31	8.4 (5.8-11.7)
Other source	27	7.3 (4.9-10.5)
Staying home	46	12.5 (9.3-16.3)
**Methods of diagnosis received**		
Microscopy	74	20.0 (16.1-24.5)
RDT	30	8.1 (5.6-11.4)
Clinical (non laboratory-based)	265	71.8 (66.9-76.4)

### Factors related to treatment-seeking

The results from interviewing key informants, public health facilities and their service were very limited in Wa State. The central government of Myanmar only runs hospitals and clinics in main towns in Wa State; they hardly provide any health service at community level. The local government of special region had a community health centre (CHC) in Gelongba. Despite there being six staff in the CHC, none of the staff had professional training in health or medicine in formal school. The only available medical instruments were two stethoscopes and three thermometers. Anti-malarial drugs had been out of stock at the time of survey. This determined why medical sellers were the most accessible and widely used health resources. Most of the medical sellers did not know precise chloroquine doses for children and dosing information they gave was often inadequate, however the patients prefer their services because they satisfy their needs. For example, even if the retailers were aware that an oral therapy would be appropriate, they might sell injectable formulations if a client asked or they knew that patients believed injections to be more effective.

The results of multivariable logistic regression analysis showed that five variables were independently associated with delayed malaria treatment. Families with an average yearly income per person more than US$200 were more likely to seek treatment for malaria within 24 hours. Households located more than 3 km from a health facility were more likely to delay seeking malaria treatment. Families, whose wives could make decisions or co-decisions, were more likely to seek treatment in time. Families were more likely to seek treatment promptly if patients were male, and for children under 15 years old (Table 3).

**Table 3:** Variables associated with delay in malaria treatment-seeking behaviour for fever patients in Gelongba and Mandong Districts, Shan Special Region II, Myanmar

	**Treatment < 24hrs (%)**	**Univariate OR (95% CI)**	**Adjusted OR (95% CI)**	**P values**
**Sex of respondents**				
Male (n=240)	76(31.8)	0.96(0.59-1.56)	0.89 (0.44-1.89)	0.936
Female (n=129)	42(32.4)	1		
**Age of respondents**				
≤ 30 (n=207)	66(32.1)	0.99(0.62-1.58)	0.96(0.37-2.06)	0.955
>30 (n=162)	52(31.9)	1		
**Educational status**				
Illiterate (n=357)	107(31.4)	0.43(0.12-1.54)	0.56(0.11-1.68)	0.345
Read and write (n=12)	11(50.0)	1		
**Average yearly income (USA)**				
≤ 100 (n=191)	34(18.0)	0.08(0.04-0.18)	0.12(0.10-0.58)	0
101-200 (n=134)	52(38.5)	0.24(0.10-0.53)	0.26(0.17-0.79)	0.002
>200 (n=44)	32(72.6)	1	1	
**Family size of the household**				
≤ 5 (n=183)	59(32.2)	1.02(0.65-1.62)	1.04 (0.46-1.89)	0.996
> 6 (n=186)	59(31.7)	1	1	
**Residence altitude**				
Altitude > 800 m (n=153)	74(48.2)	1.53(0.91-2.58)	1.15(0.67-2.72)	0.113
Altitude ≤ 800 m (n=116)	44(38.1)	1		
**Knew malaria cause**				
Yes (n=114)	34(30.2)	0.87(0.52-1.42)	0.95(0.44-1.89)	0.637
No (n=255)	84(32.8)	1	1	
**Knew malaria symptoms**				
Yes (n=261)	79(30.1)	0.776(0.47-1.27)	0.55(0.22-1.32)	0.331
No (n=108)	39(36.4)	1	1	
**Distance from health facilities**				
≤ 3 km (n=74)	44(59.8)	4.38(2.49-7.74)	2.03(1.26-6.07)	<0.0001
> 3 km (n=295)	74(25.0)	1		
**Family decision**				
Wife or co-decision (n=75)	47(62.3)	5.01(2.83-8.92)	2.65 (1.53-7.18)	<0.0001
Husband (n=283)	71(25.2)	1	1	
**Sex of fever patients**				
Male (n=227)	101(44.4)	6.79(3.62-12.9)	3.36 (1.99-13.21)	<0.0001
Female (n=142)	15(10.4)	1	1	
**Age category of fever patients**				
≤ 15 (n=41)	41(87.2)	28.7(8.57-102.02)	4.24(1.89-99.47)	<0.0001
16 – 50 (n=276)	67(24.8)	1.39(0.63-3.13)	0.71(0.24-2.56)	0.492
> 50 (n=52)	10(18.9)	1	1	

## Discussion

The health system’s deficiencies play an important role in the performance of the case management strategy of global malaria control. The political divarication between central government and local government led to the national health system (NHS) not being able to effectively cover the Wa State. Since cease-fire between the central government and local government in 1988, the central government has established three hospitals in main towns and the local government was trying to establish a health service network but is short of investment in human resources and basic facilities, and some international NGOs were running some health programmes, however all three efforts have not established an effective public health service system.

A large proportion of febrile patients sought advice or treatment, however most of them sought treatment from medical sellers, so a high proportion of the febrile was only diagnosed by clinical symptoms for malaria, and most of the microscopy or RDT were given by outreach service of NGOs. In the neighbouring region (Yunnan Province of China), 82% of malaria patients chose township community hospitals first [6]. The literature regarding treatment seeking in Myanmar and other countries of Greater Mekong subregion is rare. The situation seems similar that in sub-Saharan Africa; medicine sellers are widely used for fever and malaria treatment [14]. Despite the drug seller market being extremely informal, it is the most available and stable provider. The Roll Back Malaria (RBM) Partnership had set a target for 80% to receive appropriate treatment within 24 hours by 2010 [15]. However, this study found it was far away from that target. WHO now advocates strategies to improve home-based management of malaria, with retailer interventions being seen as one possible channel [16,17]. At present, this could be one of strategies addressing timely and appropriate treatment of malaria for the Wa people.

Wa State is an endemic area of falciparum malaria. Patients should seek treatment within 24 hours, however less than one third of patients to do so. Of 12.5% (46/369) of febrile patients who never sought treatment outside the home, 67.4% (31/46) of them sought help from supernatural spirit. In the culture of Wa ethnics, people believe in everything has its soul, so they might seek help from spirit when they are ill. A study done in Burkina Faso showed that literacy level of the heads of the households was the main factor to bring children within 24 hours to the health facility for the treatment of malaria [18]. In this study, 96.7% (357/369) of respondents were illiterate. Studies carried out in Ethiopia [19] and southern Ghana [20] showed that knowledge of respondents is not associated with malaria treatment-seeking [19]. Knowledge itself is not equal to behaviour. People’s perception, knowledge and awareness, assured material supply and enabling environment are necessary for behaviour development [21]. Family income and distances from a health facility can solve accessibility in economics and geography respectively, so the two factors are associated with treatment seeking in the study. In Wa tradition, men are usually more respectable and powerful than women [6]; on the other hand, mothers are usually child carers and housework undertakers, so wife or co-decision can increase timely treatment seeking, and male patients and children are more likely to be assisted in seeking treatment.

In the study design, the hypothesis is no difference in treatment-seeking between households in which there was someone with fever in the last two weeks compared to households without. In order to reduce recall bias, the households without fever in the last two weeks were just interviewed for socio-demographic characteristics, but for treatment-seeking and other related factors. In true-life, there may be differences in fever frequency and treatment-seeking pattern between the two household groups. This limitation may affect the results of the study. However the population of the two districts is estimated at 18, 940, but 718 households with 19.4% (3, 678) of the total population was visited. This can reduce sample bias caused by the difference.

## Conclusion

Malaria treatment-seeking behaviour is not effective, and factors affecting this include health service systems, social and cultural features in the Wa State of Myanmar.

## Competing interests

The authors declare that they have no competing interests.

## Authors’ contributions

J-WX, Q-ZX and HL designed the study and developed the protocol, analyzed and interpreted the data. HL supervised the field survey. Q-ZX, Y-R Z and HL conducted HHs survey, SDI, health facility visits and the outlet survey, and entered the data. J-WX and Q-ZX wrote the first draft of the paper. All authors read and approved the final manuscript.
